# Fluorescent organic single crystals with elastic bending flexibility: 1,4-bis(thien-2-yl)-2,3,5,6-tetrafluorobenzene derivatives

**DOI:** 10.1038/s41598-017-09848-0

**Published:** 2017-08-25

**Authors:** Shotaro Hayashi, Atsushi Asano, Natsumi Kamiya, Yoshinobu Yokomori, Takuto Maeda, Toshio Koizumi

**Affiliations:** Department of Applied Chemistry, National Defence Academy, 1-10-20 Hashirimizu, Yokosuka, 239-8686 Japan

## Abstract

Organic single crystals with elastic bending flexibility are rare because they are generally brittle. We report here fluorescent organic single crystals based on thiophene-tetrafluorobenzene-thiophene derivatives, mainly 1,4-bis(thien-2-yl)-2,3,5,6-tetrafluorobenzene. Three derivatives were synthesized by Pd-catalyzed cross-coupling reactions (Stille or direct arylation pathways). The crystallization of the derivatives gave large (mm- or cm-scale) crystals. Two crystals of 1,4-bis(thien-2-yl)-2,3,5,6-tetrafluorobenzene, **1**, and 1,4-bis(4-methylthien-2-yl)-2,3,5,6-tetrafluorobenzene, **3**, bent under applied stress and quickly recovered its original shape upon relaxation. The other crystal of 1,4-bis(5-methylthien-2-yl)-2,3,5,6-tetrafluorobenzene, **2**, showed brittle breakage under applied stress (normal behavior). Fibril lamella crystal structure based on criss-cross packed slip-stacked molecular wires and its structural integrity are important factors for the design and production of next generation crystal materials with elastic bending flexibility. Furthermore, mechanical bending–relaxation resulted in reversible change of the morphology and fluorescence (mechanofluorochromism). Such bendable crystals would lead to the next generation solid-state fluorescent and/or semiconducting materials.

## Introduction

Elastic materials such as rubbers, which are able to largely and reversibly deform, exhibit entropic elasticity and are termed as elastomers^1–3^. The materials are generally deformed by applied stress, which can lead to expanded, contracted, twisted and bent forms. The deformed shapes are quickly able to return to the original shapes. The elastomers belong to the shape-memory group of materials. Many organic materials consist of polymers^4, 5^. Most of polymers have both densely-packed (crystalline) and low-density (amorphous) domains^6^. The amorphous domains are able to disperse any stress that is applied to a material, and are able to store the external energy. The deformed materials recover their original shapes upon stress reduction, elastic deformation. The amorphous domains in the materials are thus one of the keys for the development of flexible materials^6^.

**Figure 1:**
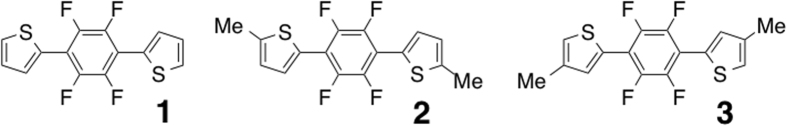
Thiophene-tetrafluorobenzene derivatives **1**, **2**, and **3**.

The most densely and regularly packed organic materials, organic single crystals, are not flexible but are hard and brittle because these have no amorphous domains. Plastically (irreversible) bending deformation of organic crystals have been discovered and studied^7–9^, and elastically (reversible) bending deformation of organic single crystals by mechanical stress have also been reported in recent years^10–13^. Thus, the organic single crystals with mechanical bending flexibility have the potential to be attractive materials but the intentional production of such bendable materials is extremely difficult. The reported organic single crystals with plastic mechanical flexibility are deformed by applied stress to give various shaped crystals. These crystals undergo irreversible deformation, as they are soft but are not elastic. The slip between crystal planes disrupts the ordered structure. Very recently, a few crystals possessing elastic bending flexibility have been discovered^10–14^. A cocrystal formed from caffeine and 4-chloro-3-nitrobenzoic acid in methanol was incidentally discovered^11^. Moreover, crystals of 2,6-dichlorobenzylidine-4-fluoro-3-nitroaniline^12^, biocrystal^13^, and terepthalamide^14^ were also discovered in recent years. Screening of enormous organic molecules may lead to accidental discoveries. We have also succeeded in obtaining the elastic organic single crystal of a fluorescent π-conjugated molecule, **3** (Fig. 1)^10^. This crystal is extremely large (up to 5 cm long) and only shows specific fluorescence (aggregation-induced enhanced emission, AIEE^15^, and mechanofluorochromism, MFC^16^) behaviour. Fibril lamella^10^ of slip-stacked (*J*-aggregated) molecular wires in the single crystal is a key morphological characteristic for the elastic crystals (it is like a fibrous organic single crystal). To advance the field of the intentional production of “elastic” and “fluorescent” organic single crystals, design of molecules based on a tetrafluorophenylene core and thienyl unit^17, 18^ is promising. Herein, we report the morphologies, optical properties and mechanical characteristics (flexibilities) of organic single crystals based on tetrafluorobenzene–thiophene derivatives.

## Results and Discussion

**Figure 2:**
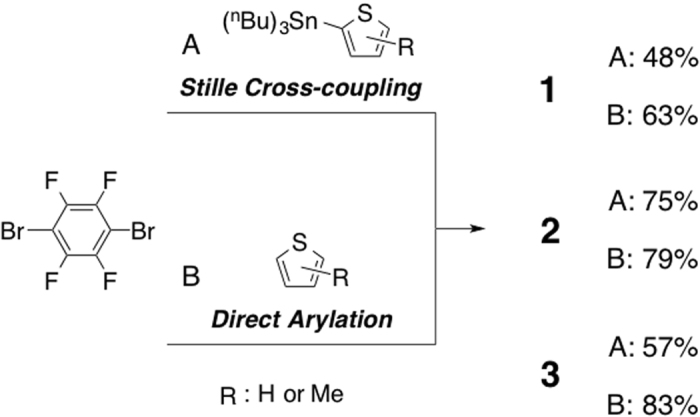
Synthesis of 1,4-bis(thien-2-yl)-2,4,5,6-tetrafluorobenzene, **1**, 1,4-bis(5-methylthien-2-yl)-2,4,5,6-tetrafluorobenzene, **2**, and 1,4-bis(4-methylthien-2-yl)-2,4,5,6-tetrafluorobenzene, **3**. (**A**) ***Stille cross-coupling***: 3.0 equivalent of 2-(tri-*n*-butyltin)thiophenes, Pd(PPh_3_)_4_, toluene, 100 °C, 24 h. (**B**) ***Direct Arylation***: 10 equivalent of thiophenes, PdCl_2_, 1AdCOOH, K_2_CO_3_, DMAc, 120 °C, 2 h.

Thiophene–tetrafluorobenzene-thiophene derivatives (**1**, **2** and **3**: Fig. 1) were synthesized by a Pd-catalyzed Stille cross-coupling reaction of 1,4-dibromo-2,3,5,6-tetrafluorobenzene with 2-(tri-*n*-butyltin)thiophene or direct (C-H) arylation reaction^19, 20^ of 1,4-dibromo-2,3,5,6-tetrafluorobenzene with thiophenes (Fig. 2). Pd-catalyzed Stille cross-coupling reaction of 1,4-dibromo-2,3,5,6-tetrafluorobenzene with 2-tri(*n*-butyl)tinthiophene derivatives gave compounds **1**, **2** and **3** in 48, 75, and 57% isolated yield (Fig. 2A). The direct arylation reaction of 1,4-dibromo-2,3,5,6-tetrafluorobenzene with thiophene derivatives gave compounds **1**, **2** and **3** in 63, 79, and 83% isolated yields, respectively (Fig. 2B). Both approaches gave the products in good yields, but direct arylation is favorable owing to the non-toxic and tin-free synthetic conditions. Crystallization of the compounds (**1**, **2** and **3**), which was optimized to give large (> mm-scale) size crystals, was performed with a biphasic methanol–dichloromethane solution containing the compounds, to yield transparent pale-yellow-colored crystal **1**, turbid yellow-colored crystal **2** and transparent pale-yellow colored crystal **3** on the millimeter- or centimeter-scale (Fig. S1).

**Figure 3:**
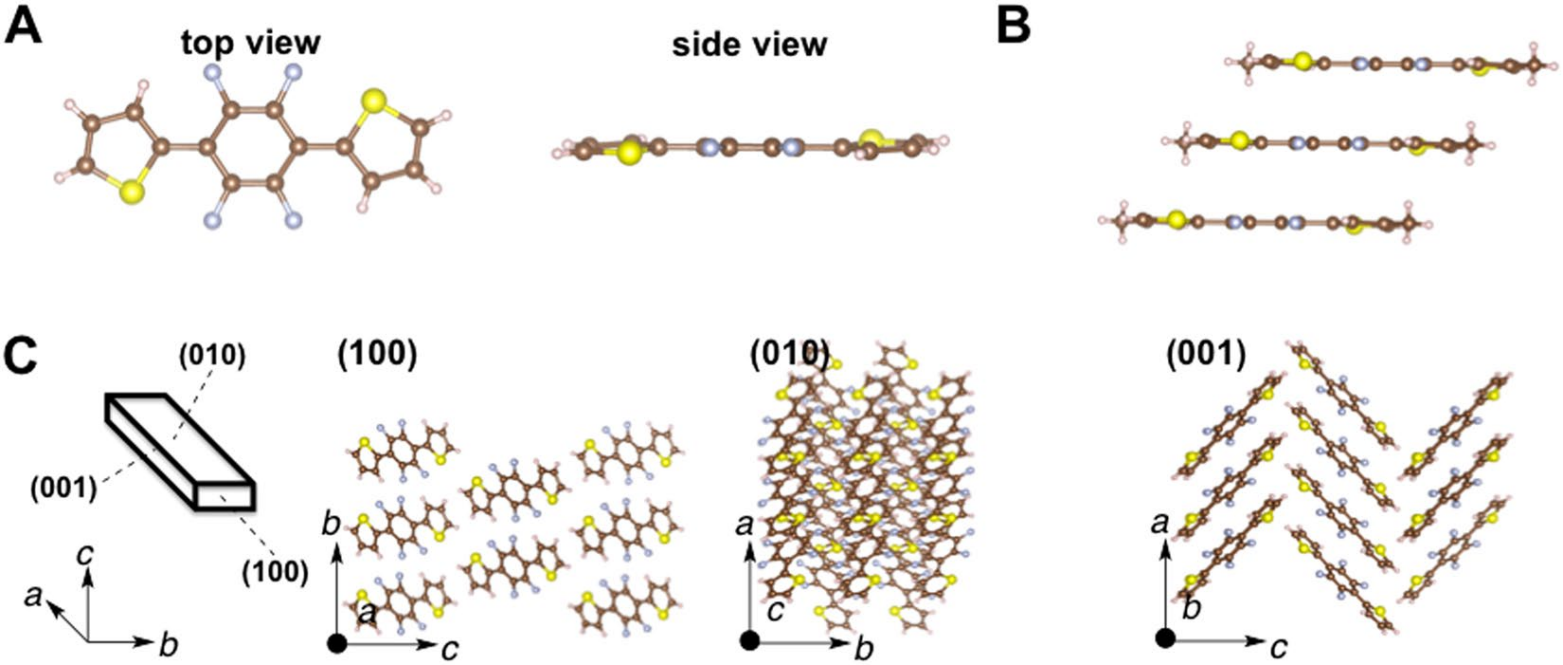
(**A**) Molecular structure (top and side views, respectively) of 1,4-bis(thienyl)-2,4,5,6-tetrafluorobenzene, **1**, in the crystal. (**B**) Slip-stacked structure, *J*-aggregate, of **1** in the crystal. (**C**) 3D crystal structure of the crystal **1**.

**Figure 4:**
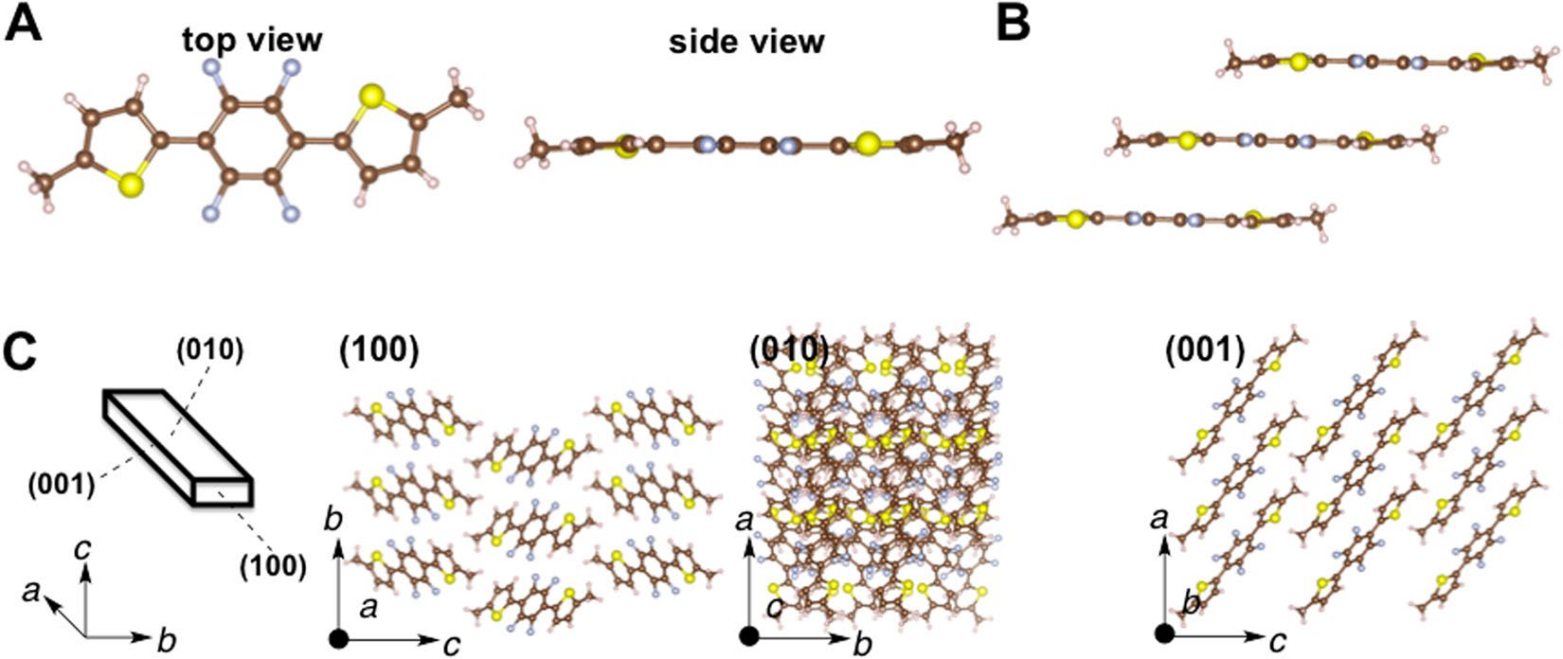
(**A**) Molecular structure (top and side views, respectively) of 1,4-bis(5-methylthienyl)-2,4,5,6-tetrafluorobenzene, **2**, in the crystal. (**B**) Slip-stacked structure, *J*-aggregate, of **2** in the crystal. (**C**) 3D crystal structure of the crystal **2**.

**Figure 5:**
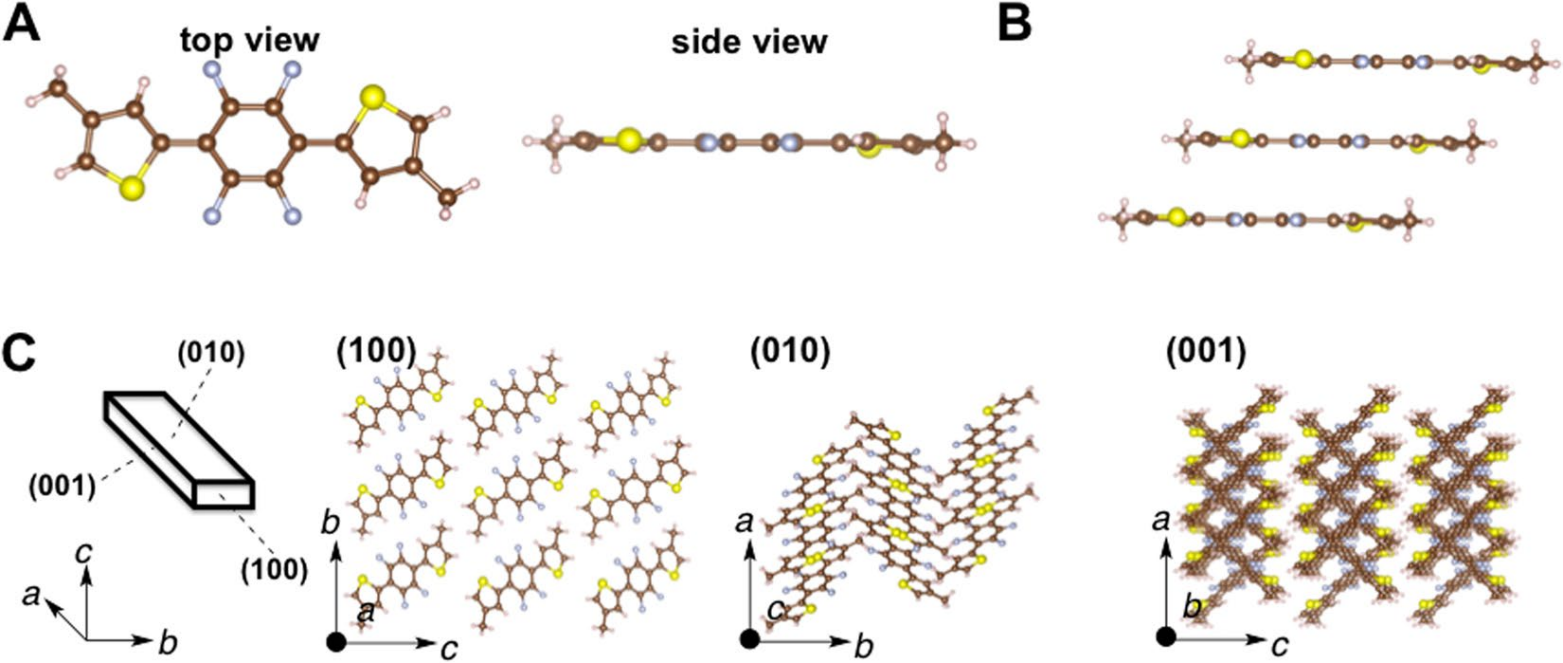
(**A**) Molecular structure (top and side views, respectively) of 1,4-bis(4-methylthienyl)-2,4,5,6-tetrafluorobenzene, **3**, in the crystal. (**B**) Slip-stacked structure, *J*-aggregate, of **3** in the crystal. (**C**) 3D crystal structure of the crystal **3**.

The crystal structure of **1** contains S–F (2.722 Å) and F–H (2.220 Å) intramolecular contacts that are significantly shorter than the sums of their van der Waals radii (*d*_SF_ = *r*_S_ + *r*_F_ = 3.27 Å, *d*_FH_ = *r*_F_ + *r*_H_ = 2.67 Å) (Fig. 3A). These results imply that intramolecular S–F and F–H interactions take place^10^. These interactions lead to the formation of highly planar molecules with a maximum torsion angle of 1.31° between the tetrafluorophenylene and thiophene units. The molecules form a slip-stacked assembly (Fig. 3B). The center-to-center separation between the thiophene–tetrafluorobenzene–thiophene planes is 2.511 Å. The fibril lamella crystal structure originates from the slip-stacked molecular wires at the (010) and (001) faces (Fig. 3C) through self-assembly of planar thiophene–tetrafluorophenylene–thiophene molecules. A criss-cross arrangement (89°) of the molecules was observed in the (001) face (Fig. 3C and Fig. S2A). The crystal structure of **2** contains S–F (2.729 Å) and F–H (2.228 Å) intramolecular contacts that are significantly shorter than the sums of their van der Waals radii (*d*_SF_ = *r*_S_ + *r*_F_ = 3.27 Å, *d*_FH_ = *r*_F_ + *r*_H_ = 2.67 Å) (Fig. 4A). These contacts result in highly planar molecules with a maximum torsion angle of 1.34° between tetrafluorophenylene and thiophene units. The molecules form a slip-stacked assembly (Fig. 4B). The center-to-center separation between the thiophene–tetrafluorobenzene–thiophene planes is 3.621 Å. The fibril lamella morphology originates from the slip-stacked molecular wires at the (010) and (001) faces (Fig. 4C) through self-assembly of planar tetrafluorophenylene–thiophene molecules. Parallel arrangement was observed in (001) face. On the other hand, the crystal structure of **3** featured S–F (2.719 Å) and F–H (2.218 Å) intramolecular contacts that were significantly shorter than the sums of their van der Waals radii [(*d*_SF_ = *r*_S_ + *r*_F_ = 3.27 Å); (*d*_FH_ = *r*_F_ + *r*_H_ = 2.67 Å)] (Fig. 5A). These contacts resulted in highly planar molecules with a maximum torsion angle of 1.27° between tetrafluorophenylene and thiophene units. The molecules formed a slip-stacked assembly. The center-to-center separation between the thiophene–tetrafluorobenzene–thiophene planes equaled 2.347 Å (Fig. 5B). The fibril lamella morphology originated from the slip-stacked molecular wires at (010) and (001) faces (Fig. 5C) through the self-assembly of planar tetrafluorophenylene–thiophene molecules. **3** also showed the morphology with the criss-cross arrangement of slip-stacked molecular wires at the (001) face similar to that of **1** (Fig. 5). The structure of **2** also showed a slip-stacked assembly (Fig. 4B), but the center-to-center separation (3.527 Å) was longer than that of **1** (2.511 Å) and **3** (2.347 Å). Moreover, parallel arrangement of the molecules was observed in the (001) face (Fig. 4C and Fig. S2B). The crystal **2** possesses a molecular structure similar to **1** and **3**, but showed a different morphology in the crystal.

Figure S3 shows the X-ray diffraction (XRD) patterns of the crystals, which were randomly set on a glass substrate. The lamella layer along the *c*-axis had an orientation of 7–10 degrees. According to the Bragg equation, the length was calculated as 11.4, 9.9 and 10.7 Å, for **1**, **2** and **3**, respectively. The layer length between the slip-stacked wires of **2** was smaller than that of **1** and **3** owing to the parallel packing of the molecules of **2** (Figs 3–5C). It is noteworthy that the (010) peak of the crystal **2** showed a higher full width at half maximum, FWHM, (0.44) than **1** (0.24) and **3** (0.22). This result suggests that the packing of **2** in the crystal is of lower quality. The lamella layer orientation of **1**, **2** and **3** along the *b*-axis was 8.9, 9.5 and 11.7 degrees, which corresponds to lengths of 9.9, 9.6 and 8.1 Å, respectively. These lamella distances are in good agreement with the crystal structure.

High resolution solid-state NMR (ssNMR) provides information on the structure and dynamics of solid materials^21–23^. The signal-to-noise ratio of the ^13^C NMR spectrum of **1** was significantly worse owing to the extremely long *T*_1_^H^ (less efficiency of cross-polarization) compared with those of **2** and **3**, indicating complete packing and a rigid molecule (Figs S4 and S5). The extremely sharp ^13^C NMR signals of **3** indicated a regularly oriented packing, while the signals of **2**, which were broader than those of **3**, implied a lower quality of packing. Similarly, a broad singlet ^19^F peak of **2** arose from the partially random packing. Every *T*_1_^F^ is very long and indicates that the benzene ring is located in very rigid circumstance. The *T*_1_^H^ of **1** was too long and we did not measure it. On the other hand, *T*_1_^H^ values of **2** and **3** were the order of several 10 s. The ^13^C NMR spectrum of **1** is very noisy because of extremely long *T*_1_^H^ (Fig. S5A). This means the CP from ^1^H to ^13^C nuclei was not efficient because the recycle delay was too short to recover for ^1^H magnetization to thermal equilibrium. The long *T*_1_^H^ is due to the rigid and complete packing of crystal **1**. The ^13^C NMR signal of **3** was very sharp, indicating regularly ordered packing (Fig. S5G). On the other hand, the broadness of **2** rather than **3** implies the less quality of packing (or existence of defects) (Fig. S5D). The ^19^F NMR spectra of **1** and **3** showed two peaks (narrow at ca. 136.5 ppm and broad at ca. 138 ppm) but that of **2** was singlet (Fig. S5B, S5E and S5H). Because of no ^1^H decoupling for ^19^F NMR measurements, the broad ^19^F peak at higher field (138 ppm) indicates the close proximity to ^1^H nuclei: the ^1^H- ^19^F dipolar interaction, which is largely influenced by the distance, makes a ^19^F signal broad (Fig. S4). There are two ^1^H- ^19^F pairs with short distance in an intra-molecule for all crystals **1** to **3**. Therefore, the appearance of two peaks suggests that the packing occurs regularly in the same direction. This means that the inter-molecular ^1^H-^19^F interaction did not occur randomly. A singlet peak at ca. 139 ppm of **2** thus results in random packing, that is, an average distance between ^1^H and ^19^F for all molecules. Actually, the center-to-center separation of **2** is longer than that of **1** and **3** (Figs 3–5B). The inter-molecular ^1^H- ^19^F interaction of **2** occurs due to closed packing between thiophene and tetrafluorobenzene units (Fig. 4B).

Thermal analyses of the crystals under nitrogen provide information of the melting point, phase transitions and crystal defects^24^. Differential scanning calorimetry (DSC) and differential thermal analysis (DTA) traces of the crystals show the melting point (Fig. S6A, S6B and Table 1). However, the trace of **2** only showed a characteristic heat energy absorption at 122 °C. If this peak arose from the impurities in the crystals, the weight loss should be observed by thermogravimetric analysis (TGA). However, TGA of **2** did not show any weight loss at approximately 122 °C (Fig. S6C). Thus, thermal analyses indicate that the crystal **2** include crystal defects. The crystal growth of **2** affords the crystal with a lower quality of packing. However, the growth of **1** and **3** crystals (mm- or cm-scale) is proposed to impart the crystals with structural integrity^25^.

**Table 1:** Physical data of compounds.

Compound	In THF (nm)		Crystal (nm)		
	λ^ab﻿﻿s﻿﻿^	λ^fl^ (Φ)	λ^ex a^	λ^fl^ (Φ)	*T*_m_^b^ (*T*_d_^c^)
**1**	323	405 (15)	415	491 (19)	170 (185)
**2**	333	407 (16)	417	499 (14)	174^d^ (201)
**3**	330	406 (15)	428	502 (25)	164 (197)

^a^Excited by 525 nm. ^b^Estimated by DSC analysis. ^c^5% Decomposition estimated by TGA analysis. ^d^The peak at 122 °C was observed.

**Figure 6:**
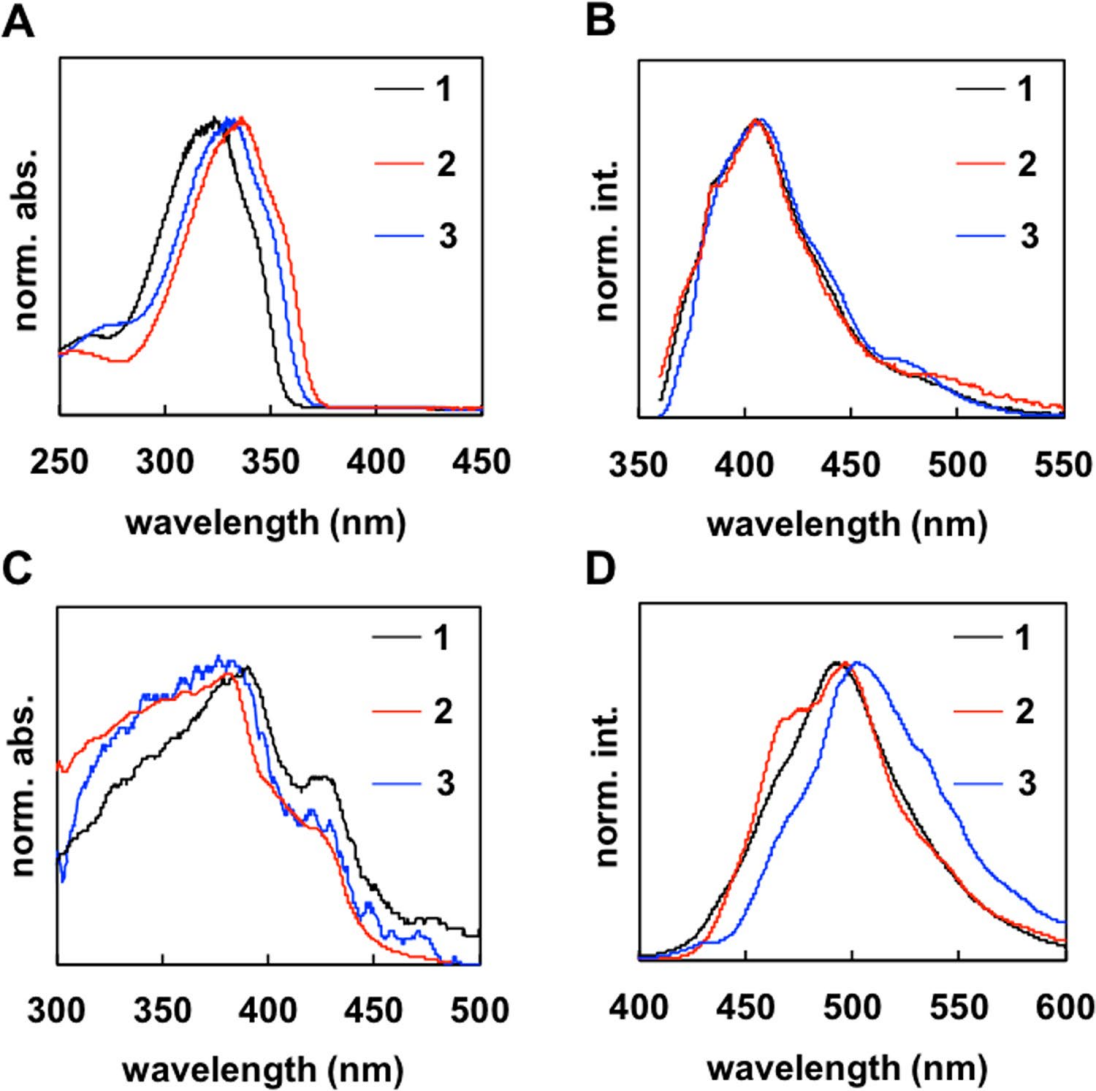
(**A**) UV-vis absorption spectra of **1**, **2** and **3** in tetrahydrofuran. (**B**) Fluorescence spectra of **1**, **2** and **3** in tetrahydrofuran. (**C**) UV-vis absorption spectra of crystals **1**, **2** and **3**. Crystal sizes are listed in the supporting information. (**D**) Fluorescence spectra of crystals **1**, **2** and **3**.

Figure 6A shows the UV-vis absorption spectra of the compounds **1**, **2** and **3** in tetrahydrofuran, THF. The absorption spectrum for **1** (λ_max_ = 323 nm, λ_edge_ = 360 nm, THF) was at a shorter wavelength compared with that of **3** (λ_max_ = 329 nm, λ_edge_ = 369 nm, THF), as a result of the hyperconjugation effect of the methyl group at the 3-position. The peak and edge of the spectrum of **2** (λ_max_ = 333 nm, λ_edge_ = 374 nm, THF) was larger than that of the others. The efficiency of the hyperconjugated linkage in **2** between the π-orbital and σ_C–H_ in the methyl group is higher than that in **3** because of “*through*” and “*cross*” conjugation, respectively. However, the fluorescence spectra of compounds **1**, **2** and **3** in THF showed similar π-π* fluorescence bands with peaks at 405–407 nm (Fig. 6B).

The absorption spectra of the crystals showed red-shifted bands at approximately 350–450 nm compared with the spectra of the compounds in THF (Fig. 6A and C). In general, a large band shift from in solution (isolate) to solid (aggregate) arises from (*i*) a planarization of the conformation and (*ii*) intermolecular interactions. The dense and regular alignment of the π-conjugated molecules brings (*i*) the restriction of the molecular motion and (*ii*) the delocalization of the π-electronic field (especially *J*-aggregation). The red-shifted absorption peaks of the crystals were proposed to arise from reason (*i*). The bands of **1** (λ_max_ = 382 nm) and **3** (λ_max_ = 386 nm) were at a slightly shorter wavelength than that of **2** (λ_max_ = 391 nm). This is also because of the hyperconjugation effect of the methyl group. The shoulder bands of the crystals at approximately 425 nm arose from reason (*ii*): *J*-aggregation of thiophene–tetrafluorobenzene–thiophene (Figs 3–5B). The fluorescence spectra of the crystals also showed shifts to lower energy compared with that in THF (Fig. 6D). This is because of the energy transfer between the molecules in the crystal. The fluorescence peak of **2** was shifted to a lower energy than that of **1** (Table 1). The peak of **3** was shifted to a lower energy compared with **1** and **2**. It is noteworthy that the spectrum of **2** showed a characteristic band at approximately 455 nm, which was also observed in the spectrum of the powder (disordered crystalline sample) **2** (Fig. S7B). This suggests that the existence of partial crystal defects expressed as a characteristic blue-shifted band for crystal **2**. The spectra of the powders (**1** and **3**) also showed the characteristic blue-shifted band (Fig. S7A), but the spectra of the crystal (**1** and **3**) did not exhibit their blue-shifted band derived from disorder.

The absolute fluorescence quantum yields (Φ, %) of the compounds in THF or crystal were measured based on an absolute method using an integrating sphere equipped with a multichannel spectrometer (Table 1). Compound **1** exhibited a lower quantum yield in THF (Φ = 15) than in the crystal (Φ = 19). Similarly, compound **3** also exhibited a lower quantum yield in THF (Φ = 15) than in the crystal (Φ = 25). These results are consistent with aggregation-induced emission (AIE) or crystallization-induced emission (CIE)^15^. The thienyl unit of the molecules undergo dynamic intramolecular rotations, which decrease the Φ values in THF. CIE and a red-shift of the fluorescence band are proposed to be caused by the restriction of intramolecular rotations (RIR) and then slip-stacking (*J*-aggregate). However, the Φ values of compound **2** were similar in both solution and as a crystal. The Φ value of the crystal **2** was lower than that of **1** and **3**. Incorporation of defects or a lower quality of packing in **2** is proposed to result in a low fluorescence efficiency and the observation of the characteristic blue-shifted band (Fig. 6D).

Flexible, anisotropic and densely packed molecular materials of delocalized π-systems (π-conjugated molecules) are highly useful compared with those with localized systems (non-conjugated molecules). These thermal stabilities and optical properties of the crystals are extremely important for the development of future elastic crystal devices.

**Figure 7:**
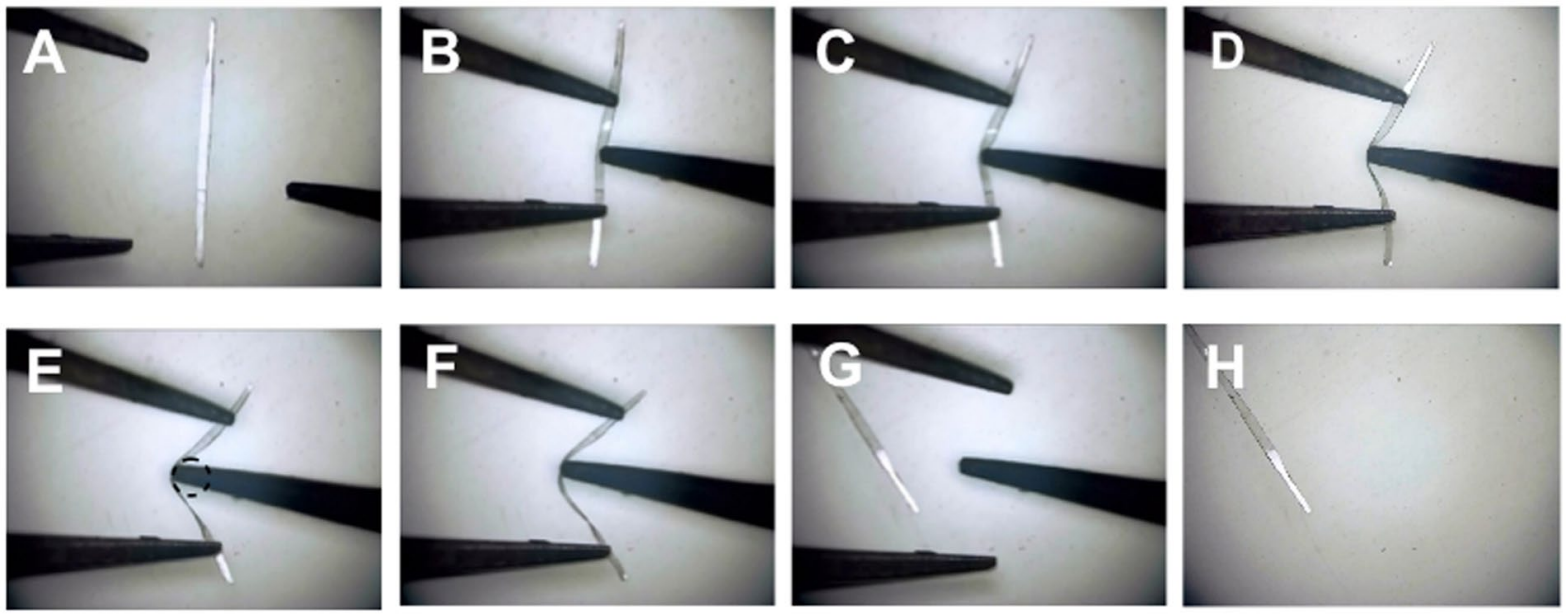
(**A**) The crystal **1** (thickness: 83 μm; width: 261 μm; length: 6.4 mm) on a glass plate. (**B**–**H**) Elastic bending motion of the crystal **1**. Stress was applied by the tips of a pair of tweezers and a needle.

Figure 7A–H and Movie S1 show the elastic behavior of a collected crystal **1** (thickness: 83 μm; width: 261 μm; length: 6.4 mm) on a glass plate. The crystal bent without breaking when it was warped. When the crystal was bent back and forth with the tips of a pair of tweezers and a needle, it adopted a bent shape (Fig. 7A–E). Upon withdrawal of the force, the bent crystal quickly recovered its original straight shape without any breaking or formation of cracks (Fig. 7F–H).

**Figure 8:**
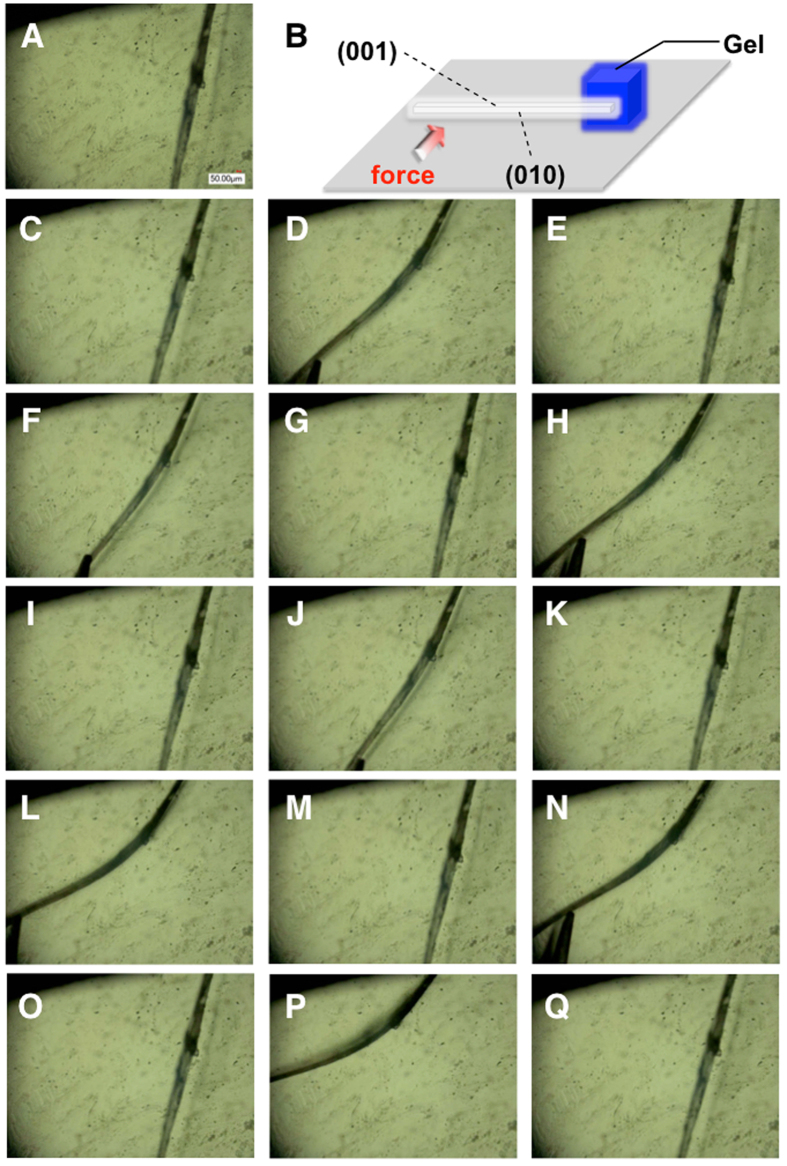
(**A**) Microscope image of the crystal **1**. (**B**) Schematic illustration of the elastic bending motion observation. (**C**–**Q**) Repeating elastic bending motion of the crystal **1**.

It is noteworthy that the crystal **1** (thickness: 98 μm; width: 191 μm; length: 7.2 mm) fixed by a gel (Fig. 8A and B) was able to exhibit an elastic bending motion many times when stress was applied by using tweezers (Fig. 8C–Q). Reversible bending–relaxation of the crystal can be cycled many times (Fig. 8 and Movie S2). The crystals (**1** and **3** ^10^) were capable of bending to the *c*-axis more than 90° under an applied stress and then quickly reverted to their original shapes upon relaxation. In contrast with the elastic bending character of the crystals **1** and **3** ^10^, the crystal **2** was brittle (Fig. S8 and Movie S3). There were no flexible crystals of **2**. The reasons for the mechanical properties are (*i*) crystal morphology and/or (*ii*) structural integrity (the existence of the crystal defects in the millimeter-scale crystal **2**) that spoil the co-operative molecular motion under the mechanical bending deformation.

We next focused on the estimation of the tension and the compression in the elastic crystal **1** (Fig. S9). Strain (*ε*_n_, %) was estimated by using the equation, *ε*_n_ = d/2r^10, 26^. The tensile ratio of the elastic crystal (ε_n_) was estimated by the original (*l*_0_), inner (*l*_in_), and outer (*l*_out_) lengths, which are illustrated in Fig. S9. To estimate the precise length of the (001) face, we observed the digital microscope image shown in Fig. 7E. The *l*_0_, *l*_in_, and *l*_out_ can be calculated from the radius of a virtual graph (circle or ellipse) on the image of the bent crystal (Fig. S9). The line tension (*dl*_tensile_) in the crystal **1** (83 μm thickness) is 41.5π. Thus, the *ε*_n_ can be estimated by the determination of the radius of circular or elliptical graphs of the bent shape crystal images. The tensile ratios (%) of the crystal **1** are calculated to be 8.1 from the graph shown in Fig. 7E.

**Figure 9:**
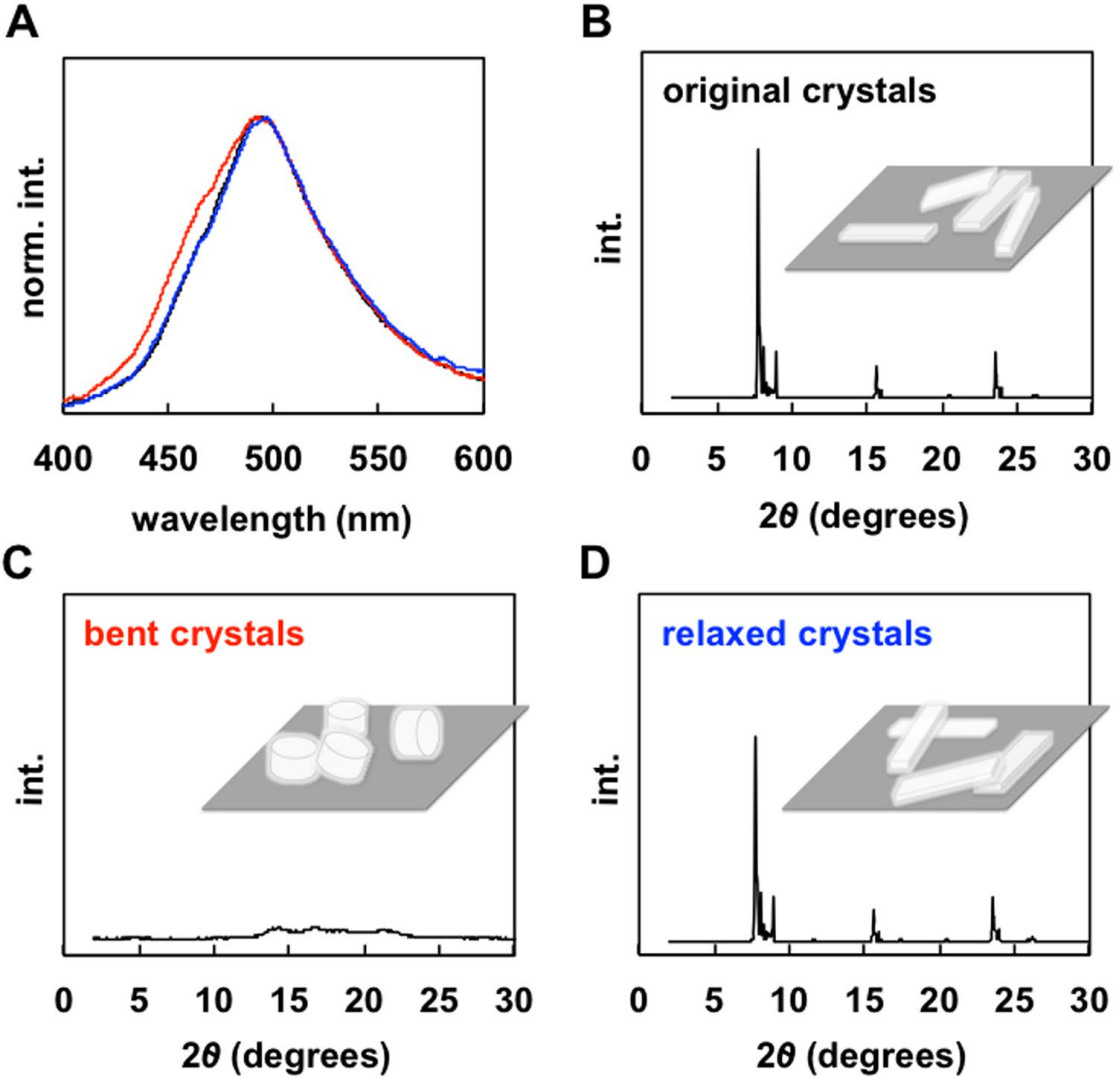
(**A**) Fluorescence spectra of the original (black line), bent (red line), and relaxed (blue line) crystals of **1**. (**B**–**D**) XRD patterns and schematic illustrations of the original (**B**), bent (**C**), and relaxed (**D**) crystals of **1**.

Fluorescence spectra and XRD analysis of the original, bent and relaxed crystals are displayed in Fig. 9. Bent crystals of **1** were prepared by (*i*) fixing both tips (100) of the original crystals using adhesive or (*ii*) fixing on the straw^10^. The bent crystals **1** exhibited a slightly high-energy fluorescence band at approximately 420–470 nm compared with the original crystals **1** (Fig. 9A). The observed fluorescence band (420–470 nm) of the crystals **1** was similar to that of the crystalline powder state of **1** (Fig. S7A). The observed blue-shifted band is proposed to arise from the slipping of the π-planes caused by bending motion^10^. The slipping (changes (expansion and contraction) of the center-to-center separation lengths)^27^ within the crystal would inhibit efficient energy transfer^28^. When the bent crystal relaxed upon force removal, the band was identical to the original spectrum. The Φ value of the bent crystals decreased to 11% compared with original crystals (19%). Relaxation of the crystals recovered the Φ value (19%). XRD patterns of these crystals were also recorded (Fig. 9B–D). The XRD patterns of the crystals of **1** showed sharp peaks in the original shape but unclear peaks in the bent shape. Upon relaxation, the XRD pattern showed sharp diffraction peaks that matched the original pattern. These results suggest that the center-to-center separation length of **1** in the slip-stacked structure would be changed under tension and compression of the crystal. The mechanofluorochromism of the crystal was observed by bending–relaxation. These reversible changes by the motion can be repeated.

It is conceivable that the crystal defects or less quality of packing in a fibril lamella spoil the cooperative molecular motion under the mechanical banding deformation. Thus, organic single crystal with structural integrity is an important factor for elastic (or plastic) mechanical deformations of densely packed materials. The crystal **2** indicates the existence of crystal defects (or less quality of packing) determined by XRD, ssNMR and DSC (or DTA) analyses (Figs S5, S6 and S7). Long-range (> mm-scale) fibril lamella morphology with structural integrity is probably required for developing elastic organic single crystal **2**. The crystallization method of π-conjugated molecule, which programed slip-stacking (*J*-aggregation) of molecule for fibril lamella morphology, is an important for developing next elastic organic single crystal.

## Conclusion

We have obtained fluorescent and elastic organic single crystals of 1,4-bis(thienyl)-2,3,5,6-tetrafluorobenzene, **1**. This crystal exhibited an elastic bending motion for many cycles when stress was applied. Fibril lamella crystal structure based on criss-cross packed slip-stacked molecular wires and their structural integrity of the crystals **1** and **3** are important factors for the design and production of next generation crystal materials. On the other hand, the crystal **2** is a brittle material, which arises from a crystal structure including partial crystal defects caused by partially random packing (lack of structural integrity). Further studies for the development of fibrous (elastic) single crystals of π-conjugated molecules and fluorescent device applications are now in progress. Moreover, we have now challenged the simulation of relationships between crystal structure and elastic bending flexibility.

## Experimental

### Chemicals

1,4-Dibromo-2,3,5,6-tetrafluorobenzene (TCI), 2-tributylstunnylthiophene (Aldrich), thiophene (Wako), 2-methylthiophene (TCI), 3-methylthiophene (TCI), 1-adamantanecarboxylic acid (TCI), K_2_CO_3_ (Kanto), palladium chloride (Wako), tetrakis(triphenylphosphine) palladium(0) (Aldrich), and dry *N*,*N*-dimethylacetoamide (Wako) were used as received. Other solvents were used as received. 2-Tributylstunnyl-4-methylthiophene and 2-tributylstunnyl-5-methylthiophene were synthesized according to the previous report^10^.

### Synthesis via Stille Cross-coupling Reaction

1,4-Dibromo-2,3,5,6-tetrafluorobenzene (307 mg, 1.00 mmol), 2-tributylstunnylthiophenes (3.00 mmol), Pd(PPh_3_)_4_ (12 mg, 6.0 μmol) were dissolved in 2 mL of dry toluene under argon. After stirred for 24 h at 100 °C, the reaction mixture was passed to silica gel (ca. 100 g) column by hexane then dichloromethane. Crystal was collected by filtration after recrystallization (dichloromethane/methanol).

### Synthesis via Direct Arylation Reaction

1,4-Dibromo-2,3,5,6-tetrafluorobenzene (307 mg, 1.00 mmol), thiophenes (1.00 mmol), 1-adamantanecarboxylic acid (16 mg, 30 mol%), K_2_CO_3_ (125 mg, 0.90 mmol) and palladium (2.0 mol%) was stirred in dry *N*,*N*-dimethylacetoamide (1.0 mL) for 2 h at 120 °C under argon. The reaction mixture was diluted by diethyl ether, rapidly cooled to room temperature, and then filtered to remove insoluble salts. A large amount of water was added to the reaction mixture, and then it was extracted with diethyl ether (5 times). The separated organic phases were dried over MgSO_4_, evaporated and dried under reduced pressure. The further purification was performed using silica gel chromatography (dichloromethane). Crystal was collected by filtration after recrystallization (dichloromethane/methanol).

Compound **1**. ^1^H NMR (300 MHz, CDCl_3_): *δ* 7.67 (*Thiophene-H*, d, 2H, *J* = 3.6), 7.56 (*Thiophene-H*, d, 2H, *J* = 5.1), 7.21 (*Thiophene-H*, t, 2H, *J* = 4.5, *J* = 8.7). ^13^C NMR (75.45 MHz, CDCl_3_): *δ* 145.7, 142.4, 130.2, 128.3, 127.8, 127.3. ^19^F NMR (254 MHz, CDCl_3_, Monofluorobenzene standard (−113.15 ppm)): *δ* −140.8. Anal. Calcd. for (C_14_H_6_F_4_S_2_): C, 53.50; H, 1.92. Found: C, 53.49; H, 1.93.

Compound **2**. ^1^H NMR (300 MHz, CDCl_3_): *δ* 7.45 (*Thiophene-H*, d, 2H, *J* = 2.1), 6.85 (*Thiophene-H*, s, 2H), 2.56 (−C*H*_3_, s, 6H). ^13^C NMR (75.45 MHz, CDCl_3_): *δ* 143.1, 142.3, 142.2, 130.3, 125.7, 125.5, 15.1. ^19^F NMR (254 MHz, CDCl_3_, Monofluorobenzene standard (−113.15 ppm)): *δ* −141.6. Anal. Calcd. for (C_16_H_10_F_4_S_2_): C, 56.13; H, 2.94. Found: C, 56.11; H, 2.94.

Compound **3**. ^1^H NMR (300 MHz, CDCl_3_): *δ* 7.46 (*Thiophene-H*, s, 2H), 7.13 (*Thiophene-H*, s, 2H), 2.38 (−C*H*_3_, s, 6H). ^13^C NMR (75.45 MHz, CDCl_3_): *δ* 145.6, 142.3, 137.9, 132.4, 127.6, 123.8, 15.6. ^19^F NMR (254 MHz, CDCl_3_, Monofluorobenzene standard (−113.15 ppm)): *δ* −141.2. Anal. Calcd. for (C_16_H_10_F_4_S_2_): C, 56.13; H, 2.94. Found: C, 56.12; H, 2.92.

## Measurements

Liquid-state ^1^H and ^13^C NMR spectra were recorded on a JEOL EX-300 spectrometer. ^19^F NMR spectra were measured using a JEOL EX-270 spectrometer. Elemental analyses were performed on a Thermo Finnigan Flash EA1112 CHN-O analyzer. UV-vis absorption spectra were obtained on an Ocean Optics USB4000-XR1 fiber spectrometer with DH2000-BAL tungsten halogen light source. Fluorescence spectra were obtained on an Ocean Optics USB4000 fiber spectrometer with PX-2 pulsed xenon light source. Absolute quantum yield was obtained by Hamamatsu C9920-02. The single crystal X-ray diffraction data for single crystals were collected at room temperature using SMART APEX II (Bruker AXS) with a CCD detector. The crystal structures were solved by the direct method and refined by full matrix least-squares using SHELXTL. XRD analysis was performed by JEOL JDX-3530 X-ray diffractometer system. Crystal images were obtained by optical microscope, KEYENCE VH-Z500R. DSC analysis was performed by a Shimadzu DS-60, which measured during heating from room temperature to 400 °C at heating rate of 10 °C/min in nitrogen. TGA analysis was performed by a Shimadzu TA-60, which measured during heating from room temperature to 600 °C at heating rate of 20 °C/min in nitrogen. High-resolution solid-state ^13^C and ^19^F nuclear magnetic resonance (NMR) spectra were measured using a Varian NMR systems 400WB spectrometer operating at 100.57 MHz for ^13^C, 376.25 MHz for ^19^F and 399.94 MHz for ^1^H. The ^13^C NMR spectra were obtained by the combined use of cross polarization (CP) from ^1^H nuclei and magic-angle spinning (MAS) with ^1^H high-power dipolar decoupling of 110 kHz under the MAS of 20 kHz. The utilized CP contact time was 2 or 4 ms and recycle delay of 200 or 500 s was used. The ^19^F MAS NMR spectra were measured using the 90 degree pulse of 1.6 μs was used under the MAS rate of 20 kHz. To obtain the ^19^F spin-lattice relaxation time (*T*_1_^F^), the conventional inversion-recovery method was employed with recycle delay of 5000 or 6000 s. ^13^C chemical shifts were measured relative to TMS (tetramethylsilane) using the methine carbon signal at 29.47 ppm for solid adamantane as an external standard. ^19^F chemical shifts were relative to CFCl_3_ referenced externally to the CF_2_ signal of Teflon at −122 ppm^29^.

## Electronic supplementary material

Supporting Information

Move S1

Movie S2

Movie S3
