# Supplementary material for: Fluorescent organic single crystals with elastic bending flexibility: 1,4-bis(thien-2-yl)-2,3,5,6-tetrafluorobenzene derivatives
Source: Sci Rep. 2017 Aug 25;7:9453. doi: 10.1038/s41598-017-09848-0 (PMC5573333; doi:10.1038/s41598-017-09848-0)
Supplement: Supplementary file 1 — Supporting Information [file 41598_2017_9848_MOESM1_ESM.doc]

Fluorescent organic single crystals with elastic bending flexibility: 1,4-bis(thien-2-yl)-2,3,5,6-tetrafluorobenzene derivatives

Shotaro Hayashi,*^a^ Atsushi Asano, ^a^ Natsumi Kamiya, ^a^ Yoshinobu Yokomori, ^a^ Takuto Maeda ^a^ and Toshio Koizumi ^a^

Department of Applied Chemistry, National Defence Academy, 1-10-20 Hashirimizu, Yokosuka, 239-8686, Japan. E-mail: shayashi@nda.ac.jp

**・Data**

**Figure S1-S7**

**Chart S1-S2**

**・Additional Files**

**Movie S1.** Elastic bending motion of crystal **1**.

**Movie S2.** Reversible elastic bending of crystal **1**.

**Movie S3.** Brittle breakage of crystal **2**.

Crystal Data of **1**

Crystal Data of **2**

Crystal Data of **3**

Fig. S1 display the photograph of the crystals under visible (A) and UV (B). UV-vis absorption spectra of the crystals (Fig. 2C) were measured by using these crystal samples. Crystal size of the samples is listed below.

Crystal **1**

thickness: 48 μm; width: 93 μm; length: 8.7 mm

Crystal **2**

thickness: 97 μm; width: 224 μm; length: 211 μm

Crystal **3**

thickness: 60 μm; width: 258 μm; length: 15.6 mm

**Figure S1**. Images of the crystals **1**, **2** and **3** under visible (A) and UV irradiation (B).

**Figure S2**. Illustration of (A) criss-cross packing and (B) parallel packing.

**Figure S3**. XRD patterns of (A) **1**, (B) **2** and (C) **3**. (D) **1**-**3** focused on (001) face. (010) face of (E) **1**, (F) **2** and (G) **3**.

**Figure S4**. The structure of the compounds. Difference of fluorine atoms (***a*** and ***b***).

**Figure S5**. High resolution solid-state ^13^C NMR spectra (A, D and G) and ^19^F NMR spectra (B, E and H) of the crystals **1**, **2**, and **3**, respectively. The plots C, F and I are the normalized ^19^F peak intensities (*M*(*t*)) obtained from the inversion-recovery method. The solid lines by the least-square fit give the ^19^F spin-lattice relaxation time (*T*_1_^F^) value; the theoretical equation is *M*(*t*)/*M*(∞) = 1 - 2∙exp (- *t*/*T*_1_^F^) and the estimated *T*_1_^F^ values are 1133 s for **1**, 817 s for **2** and 703 s for **3**, respectively.

**Figure S6**. (A) DSC trace of the crystals. (B) DTA trace of the crystals. (C) TGA analysis of the crystals.

**Figure S7**. Fluorescecne spectra of (A) **1**, (B) **2** and (C) **3** in THF (black), powder (blue) and crystal (red).

**Figure S8**. (A) Microscope image of the crystal **2**. (B-F) Brittle feature of the crystal **2**.

**Figure S9**. (A) Schematic illustration of reversible bending. (B) Straight crystal of **1**. (C, D) Bent crystals of **1**.
